# Two +ssRNA mycoviruses cohabiting the fungal cultivar of leafcutter ants

**DOI:** 10.1186/s12985-024-02465-0

**Published:** 2024-09-04

**Authors:** Asta Rødsgaard-Jørgensen, Caio Ambrosio Leal-Dutra, Sabrina Ferreira de Santana, Asger Roland Jensen, Rafael Elias Marques, Eric Roberto Guimarães Rocha Aguiar, Jonathan Zvi Shik

**Affiliations:** 1 Section for Ecology and Evolution, Department of Biology, University of Copenhagen, Universitetsparken 15, 2100 Copenhagen, Denmark; 2 Department of Biological Science, Center of Biotechnology and Genetics, Universidade Estadual de Santa Cruz, Ilhéus, Brazil; 3 Brazilian Biosciences National Laboratory (LNBio), Brazilian Center for Research in Energy and Materials (CNPEM), Campinas, Brazil; 4 Smithsonian Tropical Research Institute, Apartado Postal, 0843-03092 Balboa, Ancon, Panama

**Keywords:** Botourmiaviridae, Fungus-farming ant, Leafcutter ant, Leucoagaricus gongylophorus, Magoulivirus, Ourmia-like virus, Symbiosis, Tymovirales, Tymo-like virus, Tymovirus

## Abstract

Leafcutter ants are dominant herbivores in the Neotropics and rely on a fungus (*Leucoagaricus gongylophorus*) to transform freshly gathered leaves into a source of nourishment rather than consuming the vegetation directly. Here we report two virus-like particles that were isolated from *L. gongylophorus* and observed using transmission electron microscopy. RNA sequencing identified two +ssRNA mycovirus strains, Leucoagaricus gongylophorus tymo-like virus 1 (LgTlV1) and Leucoagaricus gongylophorus magoulivirus 1 (LgMV1). Genome annotation of LgTlV1 (7401 nt) showed conserved domains for methyltransferase, endopeptidase, viral RNA helicase, and RNA-dependent RNA polymerase (RdRp). The smaller genome of LgMV1 (2636 nt) contains one open reading frame encoding an RdRp. While we hypothesize these mycoviruses function as symbionts in leafcutter farming systems, further study will be needed to test whether they are mutualists, commensals, or parasites.

**Supplementary Information:**

The online version contains supplementary material available at 10.1186/s12985-024-02465-0.

Viruses infecting fungi (mycoviruses) appear to be common across the fungal kingdom [1]. Mycoviruses are obligately dependent on their fungal host since their inability to lyse or live outside of host cells precludes dispersal [1, 2]. Mycoviruses can be parasites that reduce host fitness, commensals that cause asymptomatic infections [3, 4], or mutualists that promote adaptive phenotypes in their hosts [5, 6]. Mycoviruses are thus likely a frontier in our understanding of fungal ecology and evolution.

Leafcutter ants depend on a fungus (*Leucoagaricus gongylophorus*) that they cultivate by provisioning with freshly cut plant fragments [7, 8]. In return, the fungus produces food reward structures for the ants, in the form of swollen hyphal cells called gongylidia. Each gongylidium contains a large vacuole that originates from an autophagic process and stores metabolites that go beyond nutritionally sustaining the ant farmers, to enzymatically breaking down freshly deposited plant material [9–11]. When leafcutter ants ingest gongylidia, they do not digest these enzymes but vector them in their fecal fluid onto the fungus garden to assist with the decomposition of newly deposited plant fragments [12, 13].

In the present study, we hypothesized that mycoviruses are common in leafcutter farming systems, given their ubiquitousness across the fungal kingdom, and could potentially have important functions. Other secondary microbial symbionts (e.g., bacteria) are present in the leafcutter farming system and promote disease resistance [14–16] or provide crucial nutritional services in ant guts [17] and in the fungus garden matrix [18]. In contrast, little is known about potential roles for mycoviruses.

Two seldom cited studies published nearly 40 years ago used transmission electron microscopy (TEM) to detect what were provisionally considered to be virus-like particles (VLPs) in *L. gongylophorus* isolated from a colony of the leafcutter ant *Acromyrmex octospinosus* [19, 20]. More recently, an in silico study using publicly available fungal transcriptomes identified potential viral sequences from the fungal cultivar isolated from an *Acromyrmex echinatior* colony (Ae332) [21, 22]. Here, we sought to first verify the presence of VLPs using TEM and then characterize the resident fungal virome using total RNA sequencing. Additionally, we sought to investigate whether leafcutter ants could potentially vector VLPs from ingested gongylidium cells back to the fungal cultivar in their fecal fluid. This could indicate that the mycoviruses can exist in an extracellular state and suggest further symbiotic functions in fungus farming systems as beneficial mutualists, costly parasites, or neutral commensals.

We first sought to visualize VLPs in axenic cultures of *L. gongylophorus* isolated from an *Atta colombica* colony (Ac2012-1) collected in a Panamanian rainforest (Table S1). We used a modified affinity chromatography method that was initially designed for isolating enveloped viruses by binding viral surface glycoconjugates to a lectin (concanavalin A), but that is also likely effective in binding other viral components in non-enveloped viruses [23, 24]. Briefly, samples were individually freeze-dried, ground in liquid nitrogen and then resuspended in Concanavalin A Sepharose 4B (Cytiva, MA, USA) buffer. We next filtered the samples, isolated VLPs with a modified version of the manufacturer’s instructions and prepared a negative control using only buffer (Supp. File S1). In addition, fecal fluid was collected from three worker ants of *A. colombica* (Ac2012-1) by pressing their abdomens with forceps and mixing the exuded liquid with 200 µL ddH_2_O. Isolated VLPs were prepared for TEM visualization on glow-discharged 200 mesh carbon-coated grids using 2% phosphotungstic acid (pH 7) as a negative stain. These samples were then examined on a CM100 BioTWIN (Phillips, Eindhoven, The Netherlands) transmission electron microscope at an accelerating voltage of 80 kV.

TEM images revealed two VLP phenotypes found in fungal isolate samples and in the fecal fluid of worker ants: (1) an icosahedral VLP of 30-nm in diameter (Fig. 1; VLP1), and (2) a bacilliform VLP 15-nm wide with lengths of ca. 30–150 nm (Fig. 1; VLP2). No VLPs were found in the negative control.

**Fig. 1:**
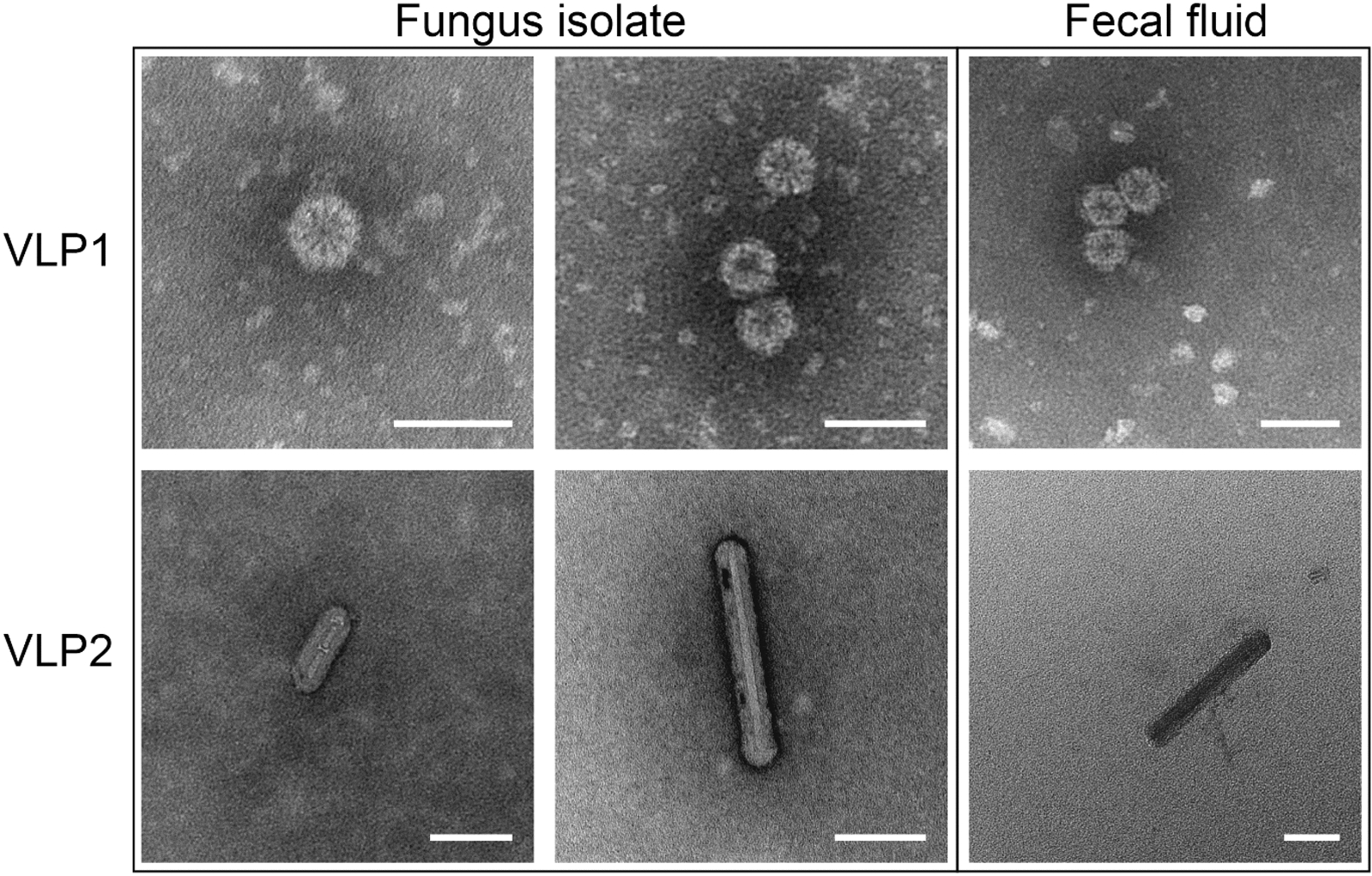
TEM images of negative stained virus-like particles (VLPs) extracted from *L. gongylophorus* fungal isolates farmed by *A. colombica* (Ac2012-1) and the fecal fluid of the worker ants from the same colony. VLP1 presents a 30-nm wide icosahedral geometry with a high similarity to known tymovirus virions [25]. VLP2 has a 15-nm wide bacilliform morphology and varies between 30 and 100 nm long like known virions of ourmiaviruses in the family *Botourmiaviridae* [26, 27]. The scale bar for all panels is 50 nm

We complemented this qualitative visualization with molecular and computational methods to identify the VLPs. We extracted total RNA from in vitro fungus cultures from the same *A. colombica* colony (Ac2012-1) with a RNeasy® Plant Mini Kit (Qiagen) using buffer RLC supplemented with DTT, following the manufacturer's protocol. Total RNA was processed for rRNA depletion, and next by 2 × 100 bp library construction using stranded cDNA (dUTP) synthesis. Library preparation and sequencing (DNBSEQ platform) were performed by BGI (Hong Kong). Low-quality reads were filtered out, and trimmed reads were provided for downstream analysis. The short reads were mapped to a high-quality draft genome from the same isolate of *L. gongylophorus* (Ac2012-1) [28] using bwa-mem2 v.2.2.1 [29]. A total of 1,323,206 unmapped read pairs were extracted with Samtools v.1.16 [30], and used for de novo RNA assembly with Trinity v.2.12.0 [31]. This generated 798 contiguous sequences.

We next used cd-hit v.4.8.1 [32] to cluster sequences with 95% similarity and filtered the dataset to remove sequences shorter than 700 nucleotides and thus limit the search for near-complete viral genomes based on the smaller genome segments in the NCBI plant and fungi virus genome database. The final dataset comprised 48 sequences ranging from 702 to 7401 nucleotides (Supp. File S2). We then identified open reading frames (ORFs) longer than 100 amino acids and translated them into amino acid sequences using Transdecoder v.5.5.0 (Haas, BJ. https://github.com/TransDecoder/TransDecoder) with default parameters (Supp. File S3). These assembled nucleotide and amino acid sequences were assessed using the Basic Local Alignment Search Tool (BLAST) against the NCBI Viral RefSeq and a custom database including all viral genomes known from fungi and plants, using BLASTn, BLASTx, and BLASTp, with both NCBI BLAST v.2.12.0+ [33] and Diamond v.2.0.13 [34] (Supp. File S1). After manually curating these sequences, we found only two viral candidate sequences of 7401 and 2636 nucleotides matching mycoviruses sequences from *Tymovirales* and *Botourmiaviridae*, respectively. These sequences are deposited on NCBI under the accessions OR914225 and OR914226, respectively. We further attempted to obtain the 5' and 3' termini of the two candidate viral sequences using 5' and 3' RACE, however, we were unsuccessful, and the terminal sequences of both viruses remain undetermined. However, the main ORFs were completely spanned by the total RNA sequencing, which allowed us to identify the main components of the viral genomes (see below).

We further mapped all reads to the viral sequences and used BEDTools v2.30.0 [35] to calculate the read count and read depth for each of them. We found a read depth of 102.17 on average for the *Tymovirales* sequence with read count of 7,615 reads while the *Botourmiaviridae* sequence had a read depth of 688.3 on average with 18,194 mapping reads (Supp. Fig. S1). Given that we used a dUTP method to sequence strand specific RNA reads, we used the mapped reads to calculate sequence polarity bias for both viral sequences. We found strong bias for positive strand in both sequences, with only two reads (0.02%) mapping to the negative strand in the *Tymovirales* sequence and 389 (2.13%) in the *Botourmiaviridae*, suggesting a low replication rate for both viruses. We then used BCFtools v1.20 [30] to call variant sites and quantify the polymorphisms in each sequence. This analysis showed that the *Tymovirales* sequence has 496 (6.7%) polymorphic sites, while the *Botourmiaviridae* has only 13 (0.49%).

To further validate the presence of the viruses, we attempted to amplify a short region (150–200 nt) of their genomes with PCR followed by Sanger sequencing. However, we only succeeded in amplifying and sequencing the sequence related to *Botourmiaviridae*, although we designed and tested 18 different primer pairs in multiple regions of the sequence related to *Tymovirales* (see Supp. File S1). While we were not able to validate the presence of the virus related to *Tymovirales* in our sample via amplification, we can assume that it maybe is present in *L. gongylophorus*, given that it was also found by Jo et al. [21] in three other samples previously sequenced by De Fine Licht et al. [22]. The high level of polymorphic sites, combined with the relatively low abundance of these sequences as indicated by the read depth and the low replication rate, likely contributed to the failure of RACE and PCR reactions.

We further assessed by BLAST the two mycovirus sequences against NCBI non-redundant viral sequences. The BLASTn analysis of the 7401 nt sequence showed high similarity to three sequences previously identified by Jo et al. [21] as Leucoagaricus tymovirus A (n = 1) and Leucoagaricus tymovirus B (n = 2) found in an isolate of *L. gongylophorus*. These sequences showed at least 95% nucleotide identity with our sequence. However, none of these three tymo-like viral sequences spanned the complete extension of our sequence (Supp. File S1, Fig. S2). The BLASTp alignment of the longest ORF showed a similar result with at least 98.2% amino acid identity to the polyprotein encoded by the same Leucoagaricus tymovirus A and B that also did not span completely our sequence (Table S2). This suggests that the three sequences found by Jo et al. [21]: (1) belong to the same viral species that we observed, (2) were more fragmented than the sequence we recovered (see Supp. File S1, Figs. S2 and S3) and (3) might be a common mycovirus across farming systems of different leafcutter ant genera as it has been found in *L. gongylophorus* from two different colonies: *Atta colombica* (this study) and *Acromyrmex echinatior* (Ae322) [21].

In contrast, BLASTn analysis of the 2636 nt sequence had a single hit to Leucoagaricus ourmiavirus F also identified from the same fungal isolate as above by Jo et al. [21] with 84% nucleotide identity, but in a very short (7%) region of our sequence. The BLASTp analysis of the longest ORF showed the highest amino acid similarity to the RNA-dependent RNA polymerase (RdRp) of Lentinula edodes magoulivirus virus 1 with 43% amino acid identity (Table S2). While this low similarity to known sequences suggests our viral sequence is an unclassified mycovirus, it still appears phylogenetically aligned with another virus found in leafcutter ant farming system.

To assess these conjectures, we next performed phylogenetic analyses for both putative viral genomes to confirm their placement among known mycoviruses. We used the amino acid sequence of the RdRp predicted from each of our genomes combined with their best hits from BLAST results as well as sequences accepted by the International Committee on Taxonomy of Viruses; (ICTV) related to *Tymovirales* or *Botourmiaviridae*. Local pairwise alignment was carried out using MAFFT v7.5 [36] and was followed by manual trimming with Aliview v1.8 [37]. We used IQ-TREE v 1.6.12 [38] to choose the best evolutionary model with the built-in ModelFinder [39] and then build the maximum likelihood trees using 1000 ultrafast bootstraps (UFBoot) [40]. Alignments are available as supplementary file S4.

We first provided support for the hypothesis that the putative tymo-like virus we sequenced belonged to the order *Tymovirales* since it grouped in a clade with, among others, Leucoagaricus tymovirus A and Leucoagaricus tymovirus B (Fig. 2A). Considering these sequences are highly similar to each other (Tables S2 and S3) and the genomic features discussed below, and considering the clade formed by them do not contain tymovirus species accepted by the ICTV (Fig. 2), we propose to reassign the strain name to “Leucoagaricus gongylophorus tymo-like virus 1” (LgTlV1) [41]. We next found support that the hypothesized magoulivirus classification was correct as our sequence grouped with maximum support (UFBoot = 100) in a clade with Lentinula edodes magoulivirus 1 (Fig. 3A). The phylogenetic tree further supported close relationships between this clade and viruses in the family *Botourmiaviridae*. Based on this evidence and the genomic structure that we describe below, we propose that our novel mycovirus be named “Leucoagaricus gongylophorus magoulivirus 1” (LgMV1).

**Fig. 2:**
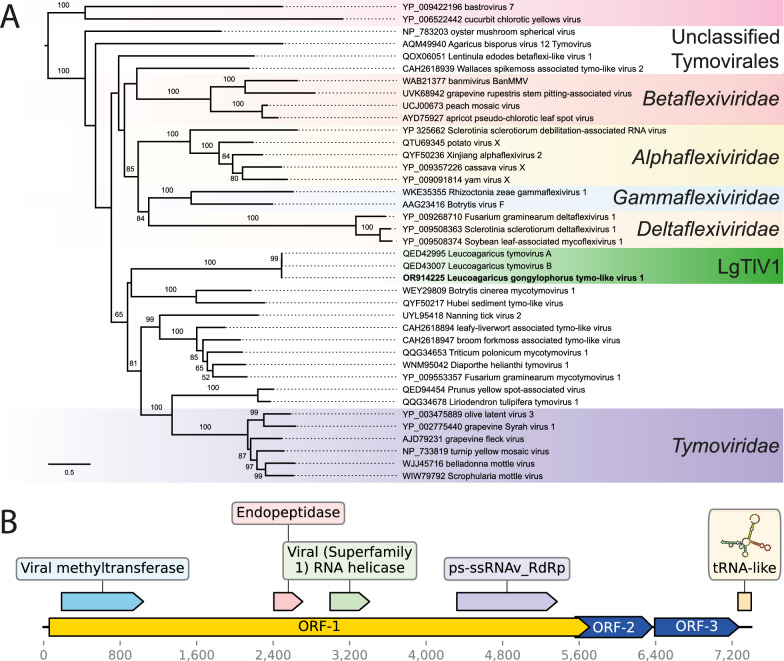
Phylogenetic placement and genomic structure of Leucoagaricus gongylophorus tymo-like virus 1 (LgTlV1) **A** maximum-likelihood phylogenetic tree based on RdRp alignment (1755 aa) showing the position of LgTlV1 (green) within the order Tymovirales. Support values on the branches are ultrafast bootstrap values calculated with 1000 replicates (only shown UFBoot ≥ 50). The scale bar indicates 0.5 amino acid substitutions per site. **B** Genome structure with ORF positions and conserved sequence regions predicted

**Fig. 3:**
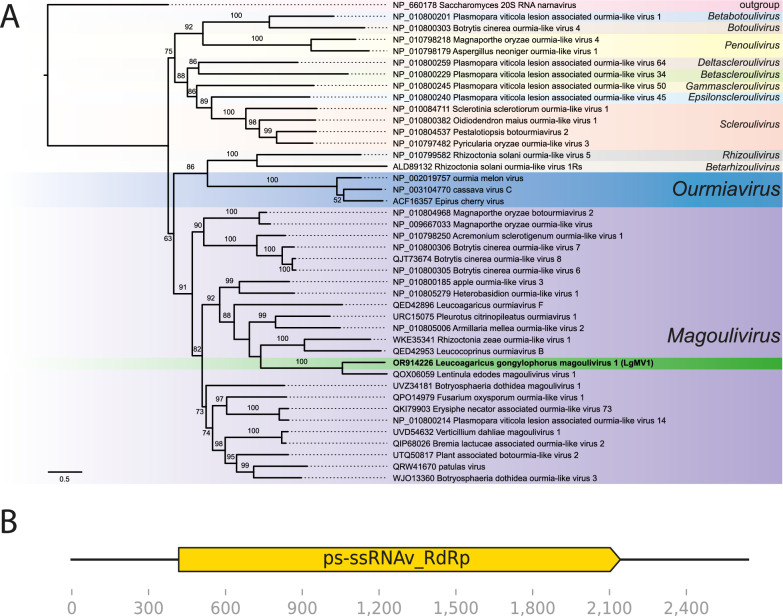
Phylogenetic placement and genomic structure of Leucoagaricus gongylophorus magoulivirus 1 (LgMV1)*.* **A** Maximum-likelihood phylogenetic tree based on RdRp alignment (1748 aa) showing the position of LgMV1 (green) within the family *Botourmiaviridae*. Support values on the branches are ultrafast bootstraps calculated with 1000 replicates (only shown UFBoot ≥ 50). The scale bar indicates 0.5 amino acid substitutions per site. **B** LgMV1 contains a single ORF that is highly similar to known RdRp sequences

We next annotated the viral genomes. Three main positive-sense ORFs were identified within the LgTlV1 genome (Fig. 2B). We annotated these ORFs using pHMMER 2.41.2 [42], InterProScan 5 v5.57–90.0 [43], and cdBlast v3.18 [44]. The largest ORF (ORF1) was located in the second reading frame and spanned 5652 nucleotides. It was predicted to encode a polyprotein containing domains with similarities to methyltransferase, endopeptidase, viral RNA helicase, and RdRp. The two other ORFs were located in the third reading frame. The smaller (ORF2) spanned 810 nucleotides and partially overlapped with ORF1. The third ORF (ORF3) spanned 885 nucleotides. No similarities to known proteins were found for ORF2 or ORF3. The annotation complements our BLAST and phylogenetic results, highlighting the consistency with other genomic studies of tymo-like viruses in terms of genome size and structure [25, 26]. These results further support that our sequence data provide a near-complete genome of Leucoagaricus gongylophorus tymo-like virus 1 (LgTlV1) relative to the ones that were previously identified, such as those of Leucoagaricus tymovirus A and Leucoagaricus tymovirus B [21], which are small and fragmented sequences of LgTlV1.

We did not identify a coat protein for LgTlV1. To investigate the possibility of a functional coat protein, we used AlphaFold v2.3.2 [45] to predict the protein structures originating from ORF2 and ORF3 and compare with the coat proteins of the related and well-characterized tymoviruses turnip yellow mosaic virus (TYMV) and belladonna mottle virus (BDMV) [25] (Fig. S4). While ORF2 and ORF3 exhibited several disordered segments, ORF3 displayed a structural central domain with the presence of alpha-helices and a beta-barrel within its protein structure (Fig. S4) reminiscent of the structure of coat proteins of TYMV and BDMV. Further investigation of this structure is needed to confirm the presence of a coat protein in LgTlV1 [46]. Using the RNAfold webserver [47], we further predicted a tRNA-like secondary structure in the final 140 nucleotides of the LgTlV1 genome (Fig. 2F). Since this feature is commonly found in plant tymoviruses [25], it reinforces their similarities and the completeness of our assembled genome sequence.

In the genome of LgMV1, we identified a positive-sense ORF spanning 1839 nucleotides. This ORF encodes a protein closely resembling a viral +ssRNA RdRp (Fig. 3B). LgMV1 resembles other species of *Botourmiaviridae* where all except ourmiaviruses are not encapsidated and have monopartite genomes encoding RdRp [26, 48]. While bacilliform virions similar to VLP2 (Fig. 1) are associated with ourmiaviruses that encode a coat protein in a separate genome segment, we did not detect such a genomic segment.

We thus cannot exclude the possibility that the observed VLP1 and VLP2 belong to LgTlV1 and LgMV1, respectively*,* or that they are other viruses we failed to detect (or perhaps not virus particles).

While the present study confirms the presence of two mycoviruses cohabiting *L. gongylophorus*, further study will be needed to test their roles as symbionts in the leafcutter ant system. We assume the mycoviruses we detected are not usually lethal pathogens, since their host leafcutter colony: (1) was likely infected since it was collected in Panama, and (2) survived in the laboratory for nearly a decade. It is of course possible that viral loads fluctuate over time and that costly parasitic functions become more pronounced if the host farming system becomes immunocompromised.

We hypothesize that, since they lack an obvious dispersal mechanism, the mycoviruses we detected are strongly dependent on the continuous health of the fungus garden, and are thus either commensals or mutualists. Detection of intact viruses in ant fecal droplets suggests that ants ingest them when they consume gongylidia and then actively vector them back to the fungus as they prepare the plant substrate. Viral vectoring would add to the list of other fungus-derived metabolites actively vectored in an undigested fully functional form back to the fungal cultivar [10, 12, 13]. Further study can explore whether the adaptive absence of active proteases in leafcutter ant digestive systems also promotes viral stability.

We propose two potential benefits of such viral stability. First, as viruses are known to trigger autophagy in their hosts [49], the identified mycoviruses may trigger autophagic processes associated with gongylidia formation [11]. Second, mycoviruses may cause the fungus to produce secondary metabolites that in turn promote antimicrobial defense. While both scenarios would support the hypothesis that the virus is a mutualist, further experimentation will be needed to test whether and how viral infection persists due to the reinoculation of the virus through the fecal fluid of ants. Potential tests of this hypothesis could include virus-curing experiments, reinfection through the fecal fluid, and subsequent measures linking viral infection and *L. gongylophorus* performance*.*

The apparent occurrence of LgTlV1 across leafcutter ant fungi farmed by other leafcutter ant genera supports both the assumption of vertical viral transmission and the potential for long-term co-evolution with this virus since earlier periods of leafcutter ant evolution. It will be important to confirm the presence of these mycoviruses in: (1) *L. gongylophorus* fungi from field-collected leafcutter ant farming systems ranging across the biogeographic distribution of leafcutter ant species from Argentina to northern Texas, and (2) the fungal pellets carried by individual dispersing leafcutter ant queens. Our findings pave the way for testing these hypotheses about symbiotic functions within leafcutter ant farming systems and highlight that the study of mycoviruses is a nascent field with potentially major biological impact.

## Supplementary Information

**Supplementary file S1:** Supplementary methods, discussion, figures and tables.

**Supplementary file S2:** Fasta file of total RNA assembly.

**Supplementary file S3:** Amino acid sequences file of identified ORFs)from total RNA.

**Supplementary file S4:** RdRp alignments used for phylogenetic reconstructions.

## Data Availability

Viral genome assemblies and RNASeq data have been uploaded to NCBI Genbank under accession numbers OR914225, OR914226, and SRR27078396 (BioProject PRJNA879936). All other data are available in the supplementary material.
