# Supplementary material for: Two +ssRNA mycoviruses cohabiting the fungal cultivar of leafcutter ants
Source: Virol J. 2024 Sep 4;21:211. doi: 10.1186/s12985-024-02465-0 (PMC11373429; doi:10.1186/s12985-024-02465-0)
Supplement: Supplementary file 1 — Supplementary file S1: Supplementary methods, discussion, figures and tables. [file 12985_2024_2465_MOESM1_ESM.pdf]

Two +ssRNA mycoviruses cohabiting the fungal cultivar of leafcutter ants

Asta Rødsgaard-Jørgensen[1*], Caio Ambrosio Leal-Dutra[1*#], Sabrina Ferreira de Santana[2], Asger Roland Jensen[1], Rafael Elias Marques[3], Eric Roberto Guimarães Rocha Aguiar[2], Jonathan Zvi Shik[1,4]

[1] Section for Ecology and Evolution, Department of Biology, University of Copenhagen, Universitetsparken 15, 2100 Copenhagen Denmark

[2] Department of Biological Science, Center of Biotechnology and Genetics, Universidade Estadual de Santa Cruz, Ilhéus, Brazil

[3] Brazilian Biosciences National Laboratory (LNBio), Brazilian Center for Research in Energy and Materials (CNPEM), Campinas, Brazil

[4] Smithsonian Tropical Research Institute, Apartado Postal 0843-03092, Balboa, Ancon, Panama

[*] These authors contributed equally to this study.

[#] **Corresponding author:** caio@bio.ku.dk

Supplementary Material Content:

Supplementary Methods
1. Culture conditions
2. Virus particle extraction
3. PCR primers and conditions
4. Identification of mycovirus sequences
5. Visualization of read depth in LgMv1 and LgTlv1

Supplementary Discussion
1. Leucoagaricus gongylophorus tymo-like virus 1 comparison

Supplementary Figure S3

Supplementary Tables
Table S1
Table S2
Table S3

Additional supplementary

34

Supplementary Methods

1. Culture condition

Leafcutter ant colonies were initially collected in Gamboa, Panama, and kept in climate-controlled rooms (25°C, 75% humidity, minimal daylight) at the University of Copenhagen. Staphylae were isolated from fungus gardens using flame-sterilized acupuncture needles and grown on Petri dishes (90 mm) containing 20-mL potato dextrose agar. From these plates, potato dextrose broth was inoculated and kept on a rotating shaker for at least 21 days at 25°C, minimal daylight. Fungi from axenic *in vitro* culture were used for further analysis.

2. Virus particle extraction

Freeze-dried fungal tissue was ground in heat-sterilized sand and 10-mL binding buffer (20mM Tris-HCl pH 7.4, 1mM CaCl2, 1mM MnCl2, 0.5M NaCl) using a mortar and pestle. The mixture was centrifuged for ten min at 16.000 g and supernatant was then filtered through syringe filters connected in this size order: 5 µm, 2.7 µm, and 0.2 µm. Concanavalin A Sepharose™ 4B (Con A Sep; Cytiva) was prepared following manufacturer's protocol and incubated with 20 mL of sample filtrate in a tumbling shaker for one hour at room temperature. After incubation, the tubes were placed vertically for ten min during which time the Con A Sep settled. The supernatant was then discarded, and the sample was mixed with 10 mL wash buffer (binding buffer, 20mM α-D-Mannopyranoside) for two min. Con A Sep was then left to settle, and supernatant was discarded. This sample was mixed with 4 mL elution buffer (20mM Tris-HCl pH 7.4, 0.5M NaCl, 0.8M α-D-Mannopyranoside) for 30 min in a tumbling shaker. The resulting eluate was collected by filtration through a syringe barrel using a 5 µm filter. Eluates were dialyzed in distilled water through D-TubeTM Dialyzer Maxi, MWCO 12-14 kDa (Novagen®) overnight and the water changed after the first two hours.

3. PCR primers and conditions

Although we attempted designing multiple primer pairs using NCBI's primer design tool (https://www.ncbi.nlm.nih.gov/tools/primer-blast/) covering several regions of the sequence, none of these worked for the *Tymovirales* sequence.

For the *Botourmiaviridae*, we first performed cDNA synthesis using iScript™ cDNA Synthesis kit (BIO-RAD) following the manufacturer's protocol. 20 µl reactions were set up by adding 4 µl 5x iScript Reaction Mix, 1 µl iScript Reverse Transcriptase, 11 µl Nuclease-free water, and

4 µl RNA template (a total of ~15.5 ng RNA) and incubated for 5 min at 25°C, 20 min at 46°C, and 1 min at 95°C. Next, we amplified the selected region using a specific primer pair designed and optimized to amplify a 164-nt region between the nt positions 1844 and 2009 (forward 5'-CACTTGGCGTGTGTTGGAAG-3'; reverse 5'-AGAGGTCCCATTTTGCCTCC-3'). To amplify the cDNA, we used a Taq DNA polymerase RED (Ampliqon) following the manufacturer instructions with an annealing temperature of 62°C. PCR products were cleaned with PureIT ExoZAP (Ampliqon, Denmark) following the manufacturer's instructions, and Sanger sequenced with the same primer pair at Eurofins (Germany).

The sequencing results validated the presence of the LgMv1 in the isolate Ac2012-1 of *L. gongylophorus*.

4. Identification of mycovirus sequences

A custom database was created from the NCBI viral genome database by retrieving RefSeq protein sequences from viral genomes with algae, plants, and fungal hosts. Assembled nucleotide and amino acid sequences from the Ac2012-1 virome were analyzed using blastx and blastp, respectively, with both NCBI Blast v.2.12.0+ [1] and Diamond v.2.0.13 [2] against the custom database. We used an e-value cutoff of 1e-3. From the blast results, the top five hits with the highest similarity to the query sequence were saved for examination of mycoviruses. The longest assembled nucleotide isoform was then chosen as a representative for each of the retrieved blast results, and subjected to NCBI blastx algorithms against all organisms, to ensure the most significant alignment. The longest ORF was identified by subjecting the longest nucleotide isoform to NCBI ORF finder (ORFfinder Viewer - NCBI (nih.gov)).

5. Visualization of read depth in LgMv1 and LgTlv1

We used the Integrative Genome Viewer (IGV) v.2.15.4 to examine the BAM files containing reads mapped to the viral sequences (Fig. S1). This allowed us to visualize the coverage and distribution of the reads across the viral genomes.

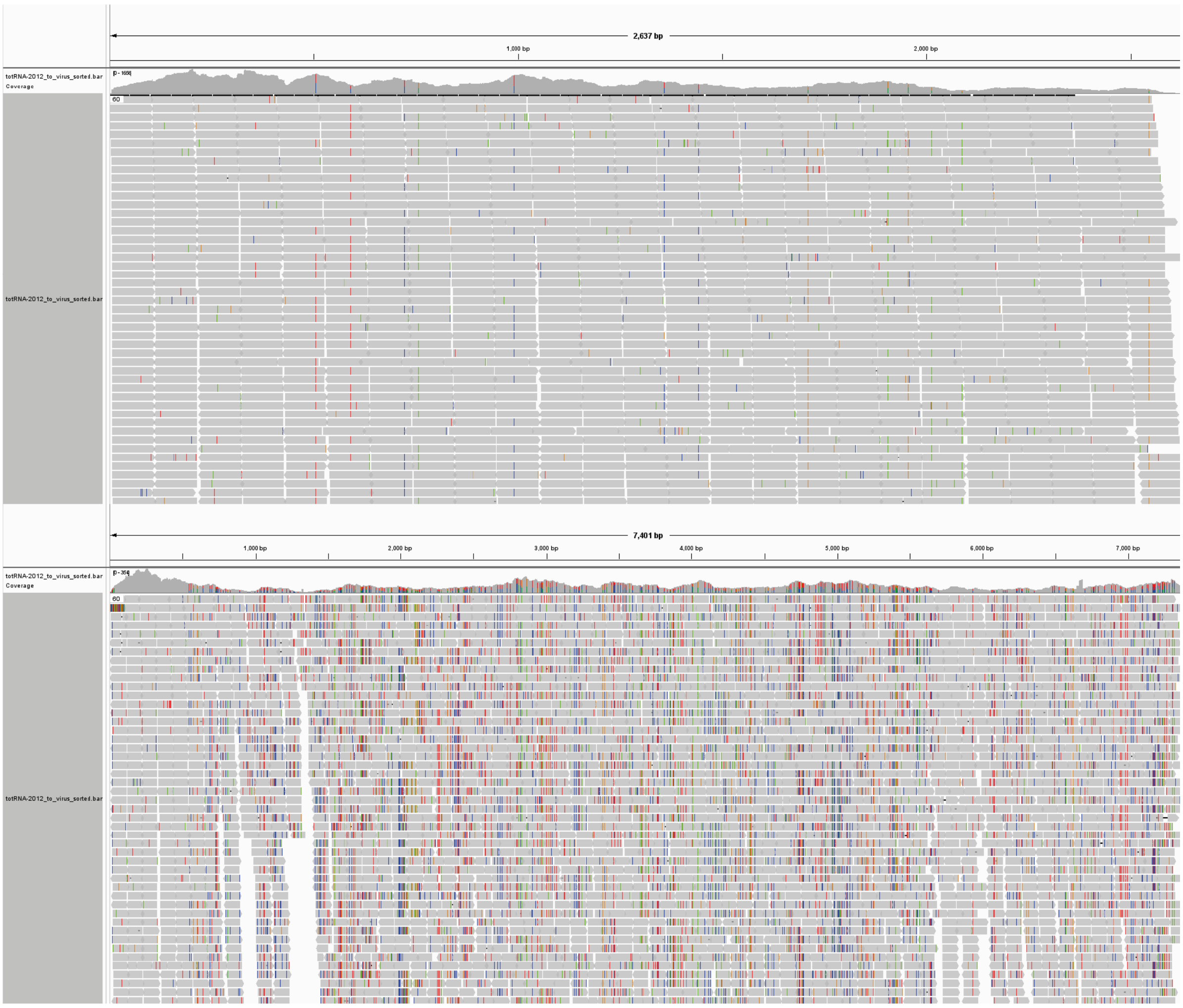

Figure S1: IGV view of read mapping coverage of the sequences generated in this study, showing LgMv1 (top) and LgTlv1 (bottom). The coverage tracks display the depth and distribution of reads mapped to each viral genome, illustrating the variation in read density across different regions of the genomes. Polymorphic sites are indicated by colored bars within the reads, highlighting the genetic variability present in the viral sequences.

Supplementary Discussion

1. Leucoagaricus gongylophorus tymo-like virus 1 comparison

In a recent study, Jo et al. [3] identified multiple mycoviruses sequences from previously RNAseq dataset publicly available in NCBI GenBank. Within these sequences, three tymo-like virus sequences were identified in the transcriptome shotgun assembly (TSA) from a *L. gongylophorus* (isolate Ae322) assembled by De Fine Licht et al. [4]. The RNA was sequenced using polyA enriched libraries and Illumina and 454 technologies.

During the transcriptomic assembly process, it is common to generate truncated or chimeric transcripts treated by the program as isoforms. This error could be responsible for the assembly of the three sequences in De Fine Licht et al. study.

When we used blastn to align the nucleotide sequences of our tymo-like virus sequence to the three “Leucoagaricus tymovirus” identified by Jo et al. (Fig. S2, Table S2, Table S3). We observed complete overlap of the three sequences to our tymo-like sequence. Additionally, the three sequences identified by Jo et al. [3] also overlap with each other in more than 1500 nucleotides (Fig. S1).

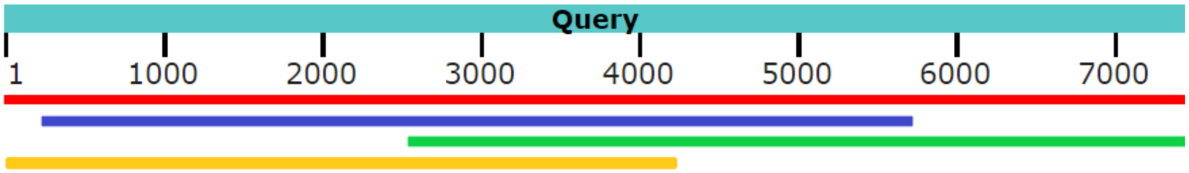

Figure S2: Blastn alignment of the sequence generated in this study (**red**) with Leucoagaricus tymo-like viruses identified previously: Leucoagaricus tymovirus B Isolate Cho (**blue**), Leucoagaricus tymovirus B Isolate Won (**green**), Leucoagaricus tymovirus A Isolate Won (**yellow**). Numbers represent nucleotide position.

We believe that the error-prone sequencing technologies, the assembly process, and virus polymorphism, might have generated several artifact sequences (referred by the assemblers as isoforms) leading to the identification of multiple similar virus sequences namely Leucoagaricus tymovirus A isolate Won, Leucoagaricus tymovirus B isolate Won, Leucoagaricus tymovirus B isolate Cho. When aligning the four sequences (Fig. S3), we found a stop codon in the sequence of isolate “Cho” which is not present in the other sequences. We believe this is a sequencing error, that is also supported by the presence of several N’s in such sequence. This suggests a low-quality sequencing that might have further introduced erroneous nucleotides likely generating false mismatches, and subsequently multiple ‘isoforms’.

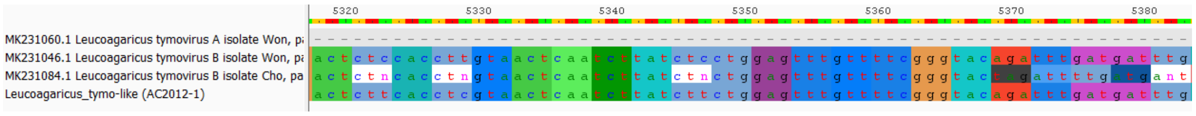

Figure S3: AliView screenshot showing a segment of the tymo-like sequences aligned. The colors represent the amino acid encoded by each codon plus stop-codon (**black**).

Supplementary Figure S3

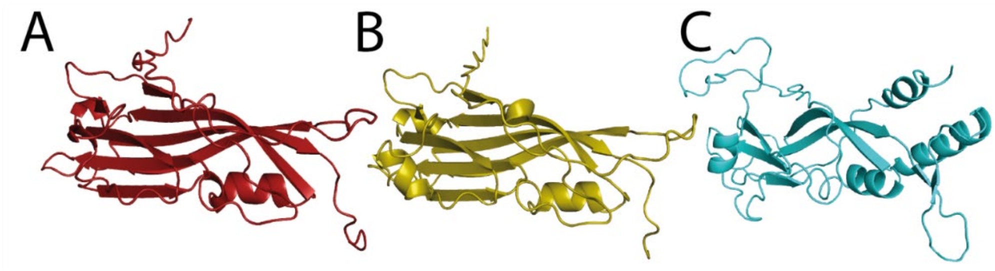

Figure S4: Predicted protein structures by AlphaFold v2.3.2 [5] from the coat proteins of turnip yellow mosaic virus (**A**), the belladonna mottle virus (**B**), and the ORF3 from Leucoagaricus gongylophorus tymo-like virus 1 (LgTlV1) (**C**). ORF-3 displayed a structural central domain with alpha helices (coils) and a beta-barrel-like structure (flat arrows) like the known coat proteins.

Supplementary Tables

Table S1. Detailed information about the colony used in this study.

Ant Species	Colony ID	Collection locality	Collection year	Collection permit
Atta colombica	Ac2012-1	Gamboa - Panama	2012	SEX/A-31-12

Table S2. Blast best hits to the identified sequences of Leucoagaricus gongylophorus tymo-like virus 1 and Leucoagaricus gongylophorus magoulivirus 1 at nucleotide and amino acid levels, against NCBI Genbank non-redundant databases.

Nucleotide

Sequence	Best hit	Q. cover	Per. Ident	Accession
**LgTlV1 Ac2012-1**	Leucoagaricus tymovirus B isolate Won	65%	96.17%	MK231046.1
	Leucoagaricus tymovirus A	56%	95.17%	MK231060.1
	Leucoagaricus tymovirus B isolate Cho	73%	95.02%	MK231084.1
**LgMV1 Ac2012-1**	Leucoagaricus ourmiavirus F isolate Won	7%	83.94%	MK231017.1

Amino acid

Sequence	Best hit	Q. cover	Per. Ident	Accession
**LgTlV1 Ac2012-1**	Leucoagaricus tymovirus B isolate Won polyprotein	56%	99.81%	QED42988.1
	Leucoagaricus tymovirus B isolate Cho polyprotein	90%	98.65%	QED43007.1
	Leucoagaricus tymovirus A polyprotein	73%	98.19%	QED42995.1
**LgMV1 Ac2012-1**	Lentinula edodes magoulivirus virus 1 RdRp	91%	46.06%	QOX06059.1
	Armillaria mellea ourmia-like virus 2 RdRp	67%	32.02%	QUD20356.1
	Rhizoctonia zeae ourmia-like virus 1 RdRp	75%	31.04%	WKE35341.1

Table S3. Pairwise percentage identity comparison between the three "Leucoagaricus tymovirus" from Jo et al. [3] at nucleotide and amino acid level.

Nucleotide full sequence

	Leucoagaricus tymovirus A	Leucoagaricus tymovirus B isolate Won	Leucoagaricus tymovirus B isolate Cho
**Leucoagaricus tymovirus A**	100%	95.72%	99.27%
**Leucoagaricus tymovirus B isolate Won**	95.72%	100%	96.61%
**Leucoagaricus tymovirus B isolate Cho**	99.27%	96.61%	100%

Amino acid Polyprotein

	Leucoagaricus tymovirus A	Leucoagaricus tymovirus B isolate Won	Leucoagaricus tymovirus B isolate Cho
**Leucoagaricus tymovirus A**	100%	98.93%	99.24%
**Leucoagaricus tymovirus B isolate Won**	98.93%	100%	99.47%
**Leucoagaricus tymovirus B isolate Cho**	99.24%	99.47%	100%

Additional supplementary

Fasta file of total RNA assembly:

Supplementary file S2 = Supp_file_S2_totalRNA_assembly.fasta

Amino acid sequences file of identified ORFs from total RNA:

Supplementary file S3 = Supp_file_S3_totalRNA_ORFS.pep

RdRp alignments used for phylogenetic reconstructions:

Supplementary file S4 = Supp_file_S4_RdRp_alignments.zip

References

1. Camacho C, Coulouris G, Avagyan V, Ma N, Papadopoulos J, Bealer K, et al. BLAST+: architecture and applications. BMC Bioinformatics. 2009;10(1):421.
2. Buchfink B, Reuter K, Drost H-G. Sensitive protein alignments at tree-of-life scale using DIAMOND. Nat Methods. 2021;18(4):366-8.
3. Jo Y, Choi H, Chu H, Cho WK. Unveiling Mycoviromes Using Fungal Transcriptomes. International Journal of Molecular Sciences. 2022;23(18):10926.
4. De Fine Licht HH, Boomsma JJ, Tunlid A. Symbiotic adaptations in the fungal cultivar of leaf-cutting ants. Nat Commun. 2014;5(1):5675.
5. Jumper J, Evans R, Pritzel A, Green T, Figurnov M, Ronneberger O, et al. Highly accurate protein structure prediction with AlphaFold. Nature. 2021;596(7873):583-9.
